# Supplementary material for: HIVprotI: an integrated web based platform for prediction and design of HIV proteins inhibitors
Source: J Cheminform. 2018 Mar 9;10:12. doi: 10.1186/s13321-018-0266-y (PMC5845081; doi:10.1186/s13321-018-0266-y)
Supplement: Supplementary file 2 — Additional file 2. Source code of HIVProtI web server. [file 13321_2018_266_MOESM2_ESM.zip › HIVprotI_Source-code/hivprotI/weka-3-6-6/doc/constant-values.html]

Constant Field Values


---


|  |  |  |
| --- | --- | --- |
| **Overview** \| Package \| Class \| **Tree** \| **Deprecated** \| **Index** \| **Help** \| **Weka's home** | |  |
| PREV   NEXT | **FRAMES**    **NO FRAMES**     **All Classes** |  |


---


# Constant Field Values


---

**Contents**

- weka.associations.\*- weka.attributeSelection.\*- weka.classifiers.\*- weka.clusterers.\*- weka.core.\*- weka.datagenerators.\*- weka.experiment.\*- weka.filters.\*- weka.gui.\*

weka.associations.\*

| weka.associations.CheckAssociator | | |
| --- | --- | --- |
| `public static final int` | `NO_CLASS` | `-1` |

| weka.associations.Tertius | | |
| --- | --- | --- |
| `public static final int` | `EXPLICIT` | `0` |
| `public static final int` | `IMPLICIT` | `1` |
| `public static final int` | `SIGNIFICANT` | `2` |

| weka.associations.tertius.Literal | | |
| --- | --- | --- |
| `public static final int` | `NEG` | `0` |
| `public static final int` | `POS` | `1` |

weka.attributeSelection.\*

| weka.attributeSelection.CostSensitiveASEvaluation | | |
| --- | --- | --- |
| `public static final int` | `MATRIX_ON_DEMAND` | `1` |
| `public static final int` | `MATRIX_SUPPLIED` | `2` |

weka.classifiers.\*

| weka.classifiers.bayes.BayesianLogisticRegression | | |
| --- | --- | --- |
| `public static final int` | `CV_BASED` | `2` |
| `public static final int` | `GAUSSIAN` | `1` |
| `public static final int` | `LAPLACIAN` | `2` |
| `public static final int` | `NORM_BASED` | `1` |
| `public static final int` | `SPECIFIC_VALUE` | `3` |

| weka.classifiers.bayes.net.search.local.Scoreable | | |
| --- | --- | --- |
| `public static final int` | `AIC` | `4` |
| `public static final int` | `BAYES` | `0` |
| `public static final int` | `BDeu` | `1` |
| `public static final int` | `ENTROPY` | `3` |
| `public static final int` | `MDL` | `2` |

| weka.classifiers.evaluation.CostCurve | | |
| --- | --- | --- |
| `public static final java.lang.String` | `NORM_EXPECTED_COST_NAME` | `"Normalized Expected Cost"` |
| `public static final java.lang.String` | `PROB_COST_FUNC_NAME` | `"Probability Cost Function"` |
| `public static final java.lang.String` | `RELATION_NAME` | `"CostCurve"` |
| `public static final java.lang.String` | `THRESHOLD_NAME` | `"Threshold"` |

| weka.classifiers.evaluation.ThresholdCurve | | |
| --- | --- | --- |
| `public static final java.lang.String` | `FALLOUT_NAME` | `"Fallout"` |
| `public static final java.lang.String` | `FALSE_NEG_NAME` | `"False Negatives"` |
| `public static final java.lang.String` | `FALSE_POS_NAME` | `"False Positives"` |
| `public static final java.lang.String` | `FMEASURE_NAME` | `"FMeasure"` |
| `public static final java.lang.String` | `FP_RATE_NAME` | `"False Positive Rate"` |
| `public static final java.lang.String` | `LIFT_NAME` | `"Lift"` |
| `public static final java.lang.String` | `PRECISION_NAME` | `"Precision"` |
| `public static final java.lang.String` | `RECALL_NAME` | `"Recall"` |
| `public static final java.lang.String` | `RELATION_NAME` | `"ThresholdCurve"` |
| `public static final java.lang.String` | `SAMPLE_SIZE_NAME` | `"Sample Size"` |
| `public static final java.lang.String` | `THRESHOLD_NAME` | `"Threshold"` |
| `public static final java.lang.String` | `TP_RATE_NAME` | `"True Positive Rate"` |
| `public static final java.lang.String` | `TRUE_NEG_NAME` | `"True Negatives"` |
| `public static final java.lang.String` | `TRUE_POS_NAME` | `"True Positives"` |

| weka.classifiers.functions.GaussianProcesses | | |
| --- | --- | --- |
| `public static final int` | `FILTER_NONE` | `2` |
| `public static final int` | `FILTER_NORMALIZE` | `0` |
| `public static final int` | `FILTER_STANDARDIZE` | `1` |

| weka.classifiers.functions.LibLINEAR | | |
| --- | --- | --- |
| `public static final int` | `SVMTYPE_L1LOSS_SVM_DUAL` | `3` |
| `public static final int` | `SVMTYPE_L2_LR` | `0` |
| `public static final int` | `SVMTYPE_L2LOSS_SVM` | `2` |
| `public static final int` | `SVMTYPE_L2LOSS_SVM_DUAL` | `1` |
| `public static final int` | `SVMTYPE_MCSVM_CS` | `4` |

| weka.classifiers.functions.LibSVM | | |
| --- | --- | --- |
| `public static final int` | `KERNELTYPE_LINEAR` | `0` |
| `public static final int` | `KERNELTYPE_POLYNOMIAL` | `1` |
| `public static final int` | `KERNELTYPE_RBF` | `2` |
| `public static final int` | `KERNELTYPE_SIGMOID` | `3` |
| `public static final int` | `SVMTYPE_C_SVC` | `0` |
| `public static final int` | `SVMTYPE_EPSILON_SVR` | `3` |
| `public static final int` | `SVMTYPE_NU_SVC` | `1` |
| `public static final int` | `SVMTYPE_NU_SVR` | `4` |
| `public static final int` | `SVMTYPE_ONE_CLASS_SVM` | `2` |

| weka.classifiers.functions.LinearRegression | | |
| --- | --- | --- |
| `public static final int` | `SELECTION_GREEDY` | `2` |
| `public static final int` | `SELECTION_M5` | `0` |
| `public static final int` | `SELECTION_NONE` | `1` |

| weka.classifiers.functions.SMO | | |
| --- | --- | --- |
| `public static final int` | `FILTER_NONE` | `2` |
| `public static final int` | `FILTER_NORMALIZE` | `0` |
| `public static final int` | `FILTER_STANDARDIZE` | `1` |

| weka.classifiers.functions.SMOreg | | |
| --- | --- | --- |
| `public static final int` | `FILTER_NONE` | `2` |
| `public static final int` | `FILTER_NORMALIZE` | `0` |
| `public static final int` | `FILTER_STANDARDIZE` | `1` |

| weka.classifiers.functions.neural.NeuralConnection | | |
| --- | --- | --- |
| `public static final int` | `CONNECTED` | `16` |
| `public static final int` | `INPUT` | `4` |
| `public static final int` | `OUTPUT` | `8` |
| `public static final int` | `PURE_INPUT` | `1` |
| `public static final int` | `PURE_OUTPUT` | `2` |
| `public static final int` | `UNCONNECTED` | `0` |

| weka.classifiers.functions.pace.MixtureDistribution | | |
| --- | --- | --- |
| `public static final int` | `NNMMethod` | `1` |
| `public static final int` | `PMMethod` | `2` |

| weka.classifiers.functions.supportVector.RegSMOImproved | | |
| --- | --- | --- |
| `public static final int` | `I0` | `3` |
| `public static final int` | `I0a` | `1` |
| `public static final int` | `I0b` | `2` |
| `public static final int` | `I1` | `4` |
| `public static final int` | `I2` | `8` |
| `public static final int` | `I3` | `16` |

| weka.classifiers.functions.supportVector.StringKernel | | |
| --- | --- | --- |
| `public static final int` | `PRUNING_LAMBDA` | `1` |
| `public static final int` | `PRUNING_NONE` | `0` |

| weka.classifiers.lazy.IBk | | |
| --- | --- | --- |
| `public static final int` | `WEIGHT_INVERSE` | `2` |
| `public static final int` | `WEIGHT_NONE` | `1` |
| `public static final int` | `WEIGHT_SIMILARITY` | `4` |

| weka.classifiers.lazy.kstar.KStarConstants | | |
| --- | --- | --- |
| `public static final int` | `B_ENTROPY` | `2` |
| `public static final int` | `B_SPHERE` | `1` |
| `public static final double` | `EPSILON` | `1.0E-5` |
| `public static final double` | `FLOOR` | `0.0` |
| `public static final double` | `FLOOR1` | `0.1` |
| `public static final double` | `INITIAL_STEP` | `0.05` |
| `public static final double` | `LOG2` | `0.693147181` |
| `public static final int` | `M_AVERAGE` | `4` |
| `public static final int` | `M_DELETE` | `1` |
| `public static final int` | `M_MAXDIFF` | `2` |
| `public static final int` | `M_NORMAL` | `3` |
| `public static final int` | `NUM_RAND_COLS` | `5` |
| `public static final int` | `OFF` | `0` |
| `public static final int` | `ON` | `1` |
| `public static final double` | `ROOT_FINDER_ACCURACY` | `0.01` |
| `public static final int` | `ROOT_FINDER_MAX_ITER` | `40` |

| weka.classifiers.meta.CostSensitiveClassifier | | |
| --- | --- | --- |
| `public static final int` | `MATRIX_ON_DEMAND` | `1` |
| `public static final int` | `MATRIX_SUPPLIED` | `2` |

| weka.classifiers.meta.GridSearch | | |
| --- | --- | --- |
| `public static final int` | `EVALUATION_ACC` | `6` |
| `public static final int` | `EVALUATION_CC` | `0` |
| `public static final int` | `EVALUATION_COMBINED` | `5` |
| `public static final int` | `EVALUATION_KAPPA` | `7` |
| `public static final int` | `EVALUATION_MAE` | `3` |
| `public static final int` | `EVALUATION_RAE` | `4` |
| `public static final int` | `EVALUATION_RMSE` | `1` |
| `public static final int` | `EVALUATION_RRSE` | `2` |
| `public static final java.lang.String` | `PREFIX_CLASSIFIER` | `"classifier."` |
| `public static final java.lang.String` | `PREFIX_FILTER` | `"filter."` |
| `public static final int` | `TRAVERSAL_BY_COLUMN` | `1` |
| `public static final int` | `TRAVERSAL_BY_ROW` | `0` |

| weka.classifiers.meta.MetaCost | | |
| --- | --- | --- |
| `public static final int` | `MATRIX_ON_DEMAND` | `1` |
| `public static final int` | `MATRIX_SUPPLIED` | `2` |

| weka.classifiers.meta.MultiClassClassifier | | |
| --- | --- | --- |
| `public static final int` | `METHOD_1_AGAINST_1` | `3` |
| `public static final int` | `METHOD_1_AGAINST_ALL` | `0` |
| `public static final int` | `METHOD_ERROR_EXHAUSTIVE` | `2` |
| `public static final int` | `METHOD_ERROR_RANDOM` | `1` |

| weka.classifiers.meta.RacedIncrementalLogitBoost | | |
| --- | --- | --- |
| `public static final int` | `PRUNETYPE_LOGLIKELIHOOD` | `1` |
| `public static final int` | `PRUNETYPE_NONE` | `0` |

| weka.classifiers.meta.ThresholdSelector | | |
| --- | --- | --- |
| `public static final int` | `ACCURACY` | `2` |
| `public static final int` | `EVAL_CROSS_VALIDATION` | `0` |
| `public static final int` | `EVAL_TRAINING_SET` | `2` |
| `public static final int` | `EVAL_TUNED_SPLIT` | `1` |
| `public static final int` | `FMEASURE` | `1` |
| `public static final int` | `OPTIMIZE_0` | `0` |
| `public static final int` | `OPTIMIZE_1` | `1` |
| `public static final int` | `OPTIMIZE_LFREQ` | `2` |
| `public static final int` | `OPTIMIZE_MFREQ` | `3` |
| `public static final int` | `OPTIMIZE_POS_NAME` | `4` |
| `public static final int` | `PRECISION` | `6` |
| `public static final int` | `RANGE_BOUNDS` | `1` |
| `public static final int` | `RANGE_NONE` | `0` |
| `public static final int` | `RECALL` | `7` |
| `public static final int` | `TP_RATE` | `5` |
| `public static final int` | `TRUE_NEG` | `4` |
| `public static final int` | `TRUE_POS` | `3` |

| weka.classifiers.meta.Vote | | |
| --- | --- | --- |
| `public static final int` | `AVERAGE_RULE` | `1` |
| `public static final int` | `MAJORITY_VOTING_RULE` | `3` |
| `public static final int` | `MAX_RULE` | `5` |
| `public static final int` | `MEDIAN_RULE` | `6` |
| `public static final int` | `MIN_RULE` | `4` |
| `public static final int` | `PRODUCT_RULE` | `2` |

| weka.classifiers.mi.MDD | | |
| --- | --- | --- |
| `public static final int` | `FILTER_NONE` | `2` |
| `public static final int` | `FILTER_NORMALIZE` | `0` |
| `public static final int` | `FILTER_STANDARDIZE` | `1` |

| weka.classifiers.mi.MIDD | | |
| --- | --- | --- |
| `public static final int` | `FILTER_NONE` | `2` |
| `public static final int` | `FILTER_NORMALIZE` | `0` |
| `public static final int` | `FILTER_STANDARDIZE` | `1` |

| weka.classifiers.mi.MIEMDD | | |
| --- | --- | --- |
| `public static final int` | `FILTER_NONE` | `2` |
| `public static final int` | `FILTER_NORMALIZE` | `0` |
| `public static final int` | `FILTER_STANDARDIZE` | `1` |

| weka.classifiers.mi.MILR | | |
| --- | --- | --- |
| `public static final int` | `ALGORITHMTYPE_ARITHMETIC` | `1` |
| `public static final int` | `ALGORITHMTYPE_DEFAULT` | `0` |
| `public static final int` | `ALGORITHMTYPE_GEOMETRIC` | `2` |

| weka.classifiers.mi.MIOptimalBall | | |
| --- | --- | --- |
| `public static final int` | `FILTER_NONE` | `2` |
| `public static final int` | `FILTER_NORMALIZE` | `0` |
| `public static final int` | `FILTER_STANDARDIZE` | `1` |

| weka.classifiers.mi.MISMO | | |
| --- | --- | --- |
| `public static final int` | `FILTER_NONE` | `2` |
| `public static final int` | `FILTER_NORMALIZE` | `0` |
| `public static final int` | `FILTER_STANDARDIZE` | `1` |

| weka.classifiers.mi.MISVM | | |
| --- | --- | --- |
| `public static final int` | `FILTER_NONE` | `2` |
| `public static final int` | `FILTER_NORMALIZE` | `0` |
| `public static final int` | `FILTER_STANDARDIZE` | `1` |

| weka.classifiers.mi.MIWrapper | | |
| --- | --- | --- |
| `public static final int` | `TESTMETHOD_ARITHMETIC` | `1` |
| `public static final int` | `TESTMETHOD_GEOMETRIC` | `2` |
| `public static final int` | `TESTMETHOD_MAXPROB` | `3` |

| weka.classifiers.mi.SimpleMI | | |
| --- | --- | --- |
| `public static final int` | `TRANSFORMMETHOD_ARITHMETIC` | `1` |
| `public static final int` | `TRANSFORMMETHOD_GEOMETRIC` | `2` |
| `public static final int` | `TRANSFORMMETHOD_MINIMAX` | `3` |

| weka.classifiers.rules.DecisionTable | | |
| --- | --- | --- |
| `public static final int` | `EVAL_ACCURACY` | `2` |
| `public static final int` | `EVAL_AUC` | `5` |
| `public static final int` | `EVAL_DEFAULT` | `1` |
| `public static final int` | `EVAL_MAE` | `4` |
| `public static final int` | `EVAL_RMSE` | `3` |

| weka.classifiers.trees.ADTree | | |
| --- | --- | --- |
| `public static final int` | `SEARCHPATH_ALL` | `0` |
| `public static final int` | `SEARCHPATH_HEAVIEST` | `1` |
| `public static final int` | `SEARCHPATH_RANDOM` | `3` |
| `public static final int` | `SEARCHPATH_ZPURE` | `2` |

| weka.classifiers.trees.BFTree | | |
| --- | --- | --- |
| `public static final int` | `PRUNING_POSTPRUNING` | `1` |
| `public static final int` | `PRUNING_PREPRUNING` | `2` |
| `public static final int` | `PRUNING_UNPRUNED` | `0` |

| weka.classifiers.trees.FT | | |
| --- | --- | --- |
| `public static final int` | `MODEL_FT` | `0` |
| `public static final int` | `MODEL_FTInner` | `2` |
| `public static final int` | `MODEL_FTLeaves` | `1` |

weka.clusterers.\*

| weka.clusterers.forOPTICSAndDBScan.DataObjects.DataObject | | |
| --- | --- | --- |
| `public static final int` | `NOISE` | `-2147483648` |
| `public static final int` | `UNCLASSIFIED` | `-1` |
| `public static final double` | `UNDEFINED` | `2.147483647E9` |

weka.core.\*

| weka.core.Attribute | | |
| --- | --- | --- |
| `public static final java.lang.String` | `ARFF_ATTRIBUTE` | `"@attribute"` |
| `public static final java.lang.String` | `ARFF_ATTRIBUTE_DATE` | `"date"` |
| `public static final java.lang.String` | `ARFF_ATTRIBUTE_INTEGER` | `"integer"` |
| `public static final java.lang.String` | `ARFF_ATTRIBUTE_NUMERIC` | `"numeric"` |
| `public static final java.lang.String` | `ARFF_ATTRIBUTE_REAL` | `"real"` |
| `public static final java.lang.String` | `ARFF_ATTRIBUTE_RELATIONAL` | `"relational"` |
| `public static final java.lang.String` | `ARFF_ATTRIBUTE_STRING` | `"string"` |
| `public static final java.lang.String` | `ARFF_END_SUBRELATION` | `"@end"` |
| `public static final int` | `DATE` | `3` |
| `public static final int` | `NOMINAL` | `1` |
| `public static final int` | `NUMERIC` | `0` |
| `public static final int` | `ORDERING_MODULO` | `2` |
| `public static final int` | `ORDERING_ORDERED` | `1` |
| `public static final int` | `ORDERING_SYMBOLIC` | `0` |
| `public static final int` | `RELATIONAL` | `4` |
| `public static final int` | `STRING` | `2` |

| weka.core.Capabilities | | |
| --- | --- | --- |
| `public static final java.lang.String` | `PROPERTIES_FILE` | `"weka/core/Capabilities.props"` |

| weka.core.ClassDiscovery | | |
| --- | --- | --- |
| `public static final boolean` | `VERBOSE` | `false` |

| weka.core.Copyright | | |
| --- | --- | --- |
| `public static final java.lang.String` | `PROPERTY_FILE` | `"weka/core/Copyright.props"` |

| weka.core.Debug.Clock | | |
| --- | --- | --- |
| `public static final int` | `FORMAT_HHMMSS` | `2` |
| `public static final int` | `FORMAT_MILLISECONDS` | `0` |
| `public static final int` | `FORMAT_SECONDS` | `1` |

| weka.core.Debug.Timestamp | | |
| --- | --- | --- |
| `public static final java.lang.String` | `DEFAULT_FORMAT` | `"yyyy-MM-dd HH:mm:ss"` |

| weka.core.Drawable | | |
| --- | --- | --- |
| `public static final int` | `BayesNet` | `2` |
| `public static final int` | `Newick` | `3` |
| `public static final int` | `NOT_DRAWABLE` | `0` |
| `public static final int` | `TREE` | `1` |

| weka.core.GlobalInfoJavadoc | | |
| --- | --- | --- |
| `public static final java.lang.String` | `GLOBALINFO_ENDTAG` | `"<!-- globalinfo-end -->"` |
| `public static final java.lang.String` | `GLOBALINFO_METHOD` | `"globalInfo"` |
| `public static final java.lang.String` | `GLOBALINFO_STARTTAG` | `"<!-- globalinfo-start -->"` |

| weka.core.Instances | | |
| --- | --- | --- |
| `public static final java.lang.String` | `ARFF_DATA` | `"@data"` |
| `public static final java.lang.String` | `ARFF_RELATION` | `"@relation"` |
| `public static final java.lang.String` | `FILE_EXTENSION` | `".arff"` |
| `public static final java.lang.String` | `SERIALIZED_OBJ_FILE_EXTENSION` | `".bsi"` |

| weka.core.Jython | | |
| --- | --- | --- |
| `public static final java.lang.String` | `CLASS_PYTHONINERPRETER` | `"org.python.util.PythonInterpreter"` |
| `public static final java.lang.String` | `CLASS_PYTHONOBJECTINPUTSTREAM` | `"org.python.util.PythonObjectInputStream"` |

| weka.core.NormalizableDistance | | |
| --- | --- | --- |
| `public static final int` | `R_MAX` | `1` |
| `public static final int` | `R_MIN` | `0` |
| `public static final int` | `R_WIDTH` | `2` |

| weka.core.OptionHandlerJavadoc | | |
| --- | --- | --- |
| `public static final java.lang.String` | `OPTIONS_ENDTAG` | `"<!-- options-end -->"` |
| `public static final java.lang.String` | `OPTIONS_STARTTAG` | `"<!-- options-start -->"` |

| weka.core.SerializationHelper | | |
| --- | --- | --- |
| `public static final java.lang.String` | `SERIAL_VERSION_UID` | `"serialVersionUID"` |

| weka.core.TechnicalInformationHandlerJavadoc | | |
| --- | --- | --- |
| `public static final java.lang.String` | `BIBTEX_ENDTAG` | `"<!-- technical-bibtex-end -->"` |
| `public static final java.lang.String` | `BIBTEX_STARTTAG` | `"<!-- technical-bibtex-start -->"` |
| `public static final java.lang.String` | `PLAINTEXT_ENDTAG` | `"<!-- technical-plaintext-end -->"` |
| `public static final java.lang.String` | `PLAINTEXT_STARTTAG` | `"<!-- technical-plaintext-start -->"` |

| weka.core.TestInstances | | |
| --- | --- | --- |
| `public static final int` | `CLASS_IS_LAST` | `-1` |
| `public static final java.lang.String` | `DEFAULT_SEPARATORS` | `" "` |
| `public static final int` | `NO_CLASS` | `-2` |

| weka.core.Version | | |
| --- | --- | --- |
| `public static final java.lang.String` | `VERSION_FILE` | `"weka/core/version.txt"` |

| weka.core.converters.Loader | | |
| --- | --- | --- |
| `public static final int` | `BATCH` | `1` |
| `public static final int` | `INCREMENTAL` | `2` |
| `public static final int` | `NONE` | `0` |

| weka.core.converters.Saver | | |
| --- | --- | --- |
| `public static final int` | `BATCH` | `1` |
| `public static final int` | `INCREMENTAL` | `2` |
| `public static final int` | `NONE` | `0` |

| weka.core.logging.Logger | | |
| --- | --- | --- |
| `public static final java.lang.String` | `PROPERTIES_FILE` | `"weka/core/logging/Logging.props"` |

| weka.core.mathematicalexpression.Scanner | | |
| --- | --- | --- |
| `public static final int` | `YYEOF` | `-1` |
| `public static final int` | `YYINITIAL` | `0` |

| weka.core.mathematicalexpression.sym | | |
| --- | --- | --- |
| `public static final int` | `ABS` | `9` |
| `public static final int` | `AND` | `29` |
| `public static final int` | `BOOLEAN` | `32` |
| `public static final int` | `CEIL` | `19` |
| `public static final int` | `COMMA` | `2` |
| `public static final int` | `COS` | `14` |
| `public static final int` | `DIVISION` | `8` |
| `public static final int` | `EOF` | `0` |
| `public static final int` | `EQ` | `27` |
| `public static final int` | `error` | `1` |
| `public static final int` | `EXP` | `12` |
| `public static final int` | `FALSE` | `22` |
| `public static final int` | `FLOOR` | `17` |
| `public static final int` | `GE` | `26` |
| `public static final int` | `GT` | `25` |
| `public static final int` | `IFELSE` | `20` |
| `public static final int` | `LE` | `24` |
| `public static final int` | `LOG` | `11` |
| `public static final int` | `LPAREN` | `3` |
| `public static final int` | `LT` | `23` |
| `public static final int` | `MINUS` | `5` |
| `public static final int` | `NOT` | `28` |
| `public static final int` | `NUMBER` | `31` |
| `public static final int` | `OR` | `30` |
| `public static final int` | `PLUS` | `6` |
| `public static final int` | `POW` | `18` |
| `public static final int` | `RINT` | `16` |
| `public static final int` | `RPAREN` | `4` |
| `public static final int` | `SIN` | `13` |
| `public static final int` | `SQRT` | `10` |
| `public static final int` | `TAN` | `15` |
| `public static final int` | `TIMES` | `7` |
| `public static final int` | `TRUE` | `21` |
| `public static final int` | `VARIABLE` | `33` |

| weka.core.matrix.Maths | | |
| --- | --- | --- |
| `public static final int` | `chisqDistribution` | `2` |
| `public static final double` | `logPSI` | `-0.9189385332046727` |
| `public static final int` | `normalDistribution` | `1` |
| `public static final double` | `PSI` | `0.3989422804014327` |
| `public static final int` | `undefinedDistribution` | `0` |

| weka.core.neighboursearch.KDTree | | |
| --- | --- | --- |
| `public static final int` | `MAX` | `1` |
| `public static final int` | `MIN` | `0` |
| `public static final int` | `WIDTH` | `2` |

| weka.core.neighboursearch.kdtrees.KDTreeNodeSplitter | | |
| --- | --- | --- |
| `public static final int` | `MAX` | `1` |
| `public static final int` | `MIN` | `0` |
| `public static final int` | `WIDTH` | `2` |

| weka.core.pmml.MappingInfo | | |
| --- | --- | --- |
| `public static final int` | `UNKNOWN_NOMINAL_VALUE` | `-1` |

| weka.core.stemmers.SnowballStemmer | | |
| --- | --- | --- |
| `public static final java.lang.String` | `PACKAGE` | `"org.tartarus.snowball"` |
| `public static final java.lang.String` | `PACKAGE_EXT` | `"org.tartarus.snowball.ext"` |

| weka.core.xml.KOML | | |
| --- | --- | --- |
| `public static final java.lang.String` | `FILE_EXTENSION` | `".koml"` |

| weka.core.xml.XMLBasicSerialization | | |
| --- | --- | --- |
| `public static final java.lang.String` | `VAL_CELLS` | `"cells"` |
| `public static final java.lang.String` | `VAL_KEY` | `"key"` |
| `public static final java.lang.String` | `VAL_MAPPING` | `"mapping"` |
| `public static final java.lang.String` | `VAL_VALUE` | `"value"` |

| weka.core.xml.XMLDocument | | |
| --- | --- | --- |
| `public static final java.lang.String` | `ATT_NAME` | `"name"` |
| `public static final java.lang.String` | `ATT_VERSION` | `"version"` |
| `public static final java.lang.String` | `DTD_ANY` | `"ANY"` |
| `public static final java.lang.String` | `DTD_AT_LEAST_ONE` | `"+"` |
| `public static final java.lang.String` | `DTD_ATTLIST` | `"ATTLIST"` |
| `public static final java.lang.String` | `DTD_CDATA` | `"CDATA"` |
| `public static final java.lang.String` | `DTD_DOCTYPE` | `"DOCTYPE"` |
| `public static final java.lang.String` | `DTD_ELEMENT` | `"ELEMENT"` |
| `public static final java.lang.String` | `DTD_IMPLIED` | `"#IMPLIED"` |
| `public static final java.lang.String` | `DTD_OPTIONAL` | `"?"` |
| `public static final java.lang.String` | `DTD_PCDATA` | `"#PCDATA"` |
| `public static final java.lang.String` | `DTD_REQUIRED` | `"#REQUIRED"` |
| `public static final java.lang.String` | `DTD_SEPARATOR` | `"\|"` |
| `public static final java.lang.String` | `DTD_ZERO_OR_MORE` | `"*"` |
| `public static final java.lang.String` | `PI` | `"<?xml version=\"1.0\" encoding=\"utf-8\"?>"` |
| `public static final java.lang.String` | `VAL_NO` | `"no"` |
| `public static final java.lang.String` | `VAL_YES` | `"yes"` |

| weka.core.xml.XMLInstances | | |
| --- | --- | --- |
| `public static final java.lang.String` | `ATT_CLASS` | `"class"` |
| `public static final java.lang.String` | `ATT_FORMAT` | `"format"` |
| `public static final java.lang.String` | `ATT_INDEX` | `"index"` |
| `public static final java.lang.String` | `ATT_MISSING` | `"missing"` |
| `public static final java.lang.String` | `ATT_TYPE` | `"type"` |
| `public static final java.lang.String` | `ATT_VERSION` | `"version"` |
| `public static final java.lang.String` | `ATT_WEIGHT` | `"weight"` |
| `public static final java.lang.String` | `TAG_ATTRIBUTE` | `"attribute"` |
| `public static final java.lang.String` | `TAG_ATTRIBUTES` | `"attributes"` |
| `public static final java.lang.String` | `TAG_BODY` | `"body"` |
| `public static final java.lang.String` | `TAG_DATASET` | `"dataset"` |
| `public static final java.lang.String` | `TAG_HEADER` | `"header"` |
| `public static final java.lang.String` | `TAG_INSTANCE` | `"instance"` |
| `public static final java.lang.String` | `TAG_INSTANCES` | `"instances"` |
| `public static final java.lang.String` | `TAG_LABEL` | `"label"` |
| `public static final java.lang.String` | `TAG_LABELS` | `"labels"` |
| `public static final java.lang.String` | `TAG_METADATA` | `"metadata"` |
| `public static final java.lang.String` | `TAG_NOTES` | `"notes"` |
| `public static final java.lang.String` | `TAG_PROPERTY` | `"property"` |
| `public static final java.lang.String` | `TAG_VALUE` | `"value"` |
| `public static final java.lang.String` | `VAL_DATE` | `"date"` |
| `public static final java.lang.String` | `VAL_NOMINAL` | `"nominal"` |
| `public static final java.lang.String` | `VAL_NORMAL` | `"normal"` |
| `public static final java.lang.String` | `VAL_NUMERIC` | `"numeric"` |
| `public static final java.lang.String` | `VAL_RELATIONAL` | `"relational"` |
| `public static final java.lang.String` | `VAL_SPARSE` | `"sparse"` |
| `public static final java.lang.String` | `VAL_STRING` | `"string"` |

| weka.core.xml.XMLOptions | | |
| --- | --- | --- |
| `public static final java.lang.String` | `ATT_NAME` | `"name"` |
| `public static final java.lang.String` | `ATT_TYPE` | `"type"` |
| `public static final java.lang.String` | `ATT_VALUE` | `"value"` |
| `public static final java.lang.String` | `DOCTYPE` | `"<!DOCTYPE options\n[\n <!ELEMENT options (option)*>\n <!ATTLIST options type CDATA \"optionhandler\">\n <!ATTLIST options value CDATA \"\">\n <!ELEMENT option (#PCDATA \| options)*>\n <!ATTLIST option name CDATA #REQUIRED>\n <!ATTLIST option type (flag \| single \| hyphens \| quotes) \"single\">\n]\n>"` |
| `public static final java.lang.String` | `ROOT_NODE` | `"options"` |
| `public static final java.lang.String` | `TAG_OPTION` | `"option"` |
| `public static final java.lang.String` | `TAG_OPTIONS` | `"options"` |
| `public static final java.lang.String` | `VAL_TYPE_CLASSIFIER` | `"classifier"` |
| `public static final java.lang.String` | `VAL_TYPE_FLAG` | `"flag"` |
| `public static final java.lang.String` | `VAL_TYPE_HYPHENS` | `"hyphens"` |
| `public static final java.lang.String` | `VAL_TYPE_OPTIONHANDLER` | `"optionhandler"` |
| `public static final java.lang.String` | `VAL_TYPE_QUOTES` | `"quotes"` |
| `public static final java.lang.String` | `VAL_TYPE_SINGLE` | `"single"` |

| weka.core.xml.XMLSerialization | | |
| --- | --- | --- |
| `public static final java.lang.String` | `ATT_ARRAY` | `"array"` |
| `public static final java.lang.String` | `ATT_ARRAY_DEFAULT` | `"no"` |
| `public static final java.lang.String` | `ATT_CLASS` | `"class"` |
| `public static final java.lang.String` | `ATT_NAME` | `"name"` |
| `public static final java.lang.String` | `ATT_NULL` | `"null"` |
| `public static final java.lang.String` | `ATT_NULL_DEFAULT` | `"no"` |
| `public static final java.lang.String` | `ATT_PRIMITIVE` | `"primitive"` |
| `public static final java.lang.String` | `ATT_PRIMITIVE_DEFAULT` | `"no"` |
| `public static final java.lang.String` | `ATT_VERSION` | `"version"` |
| `public static final java.lang.String` | `ROOT_NODE` | `"object"` |
| `public static final java.lang.String` | `TAG_OBJECT` | `"object"` |
| `public static final java.lang.String` | `VAL_NO` | `"no"` |
| `public static final java.lang.String` | `VAL_ROOT` | `"__root__"` |
| `public static final java.lang.String` | `VAL_YES` | `"yes"` |

| weka.core.xml.XStream | | |
| --- | --- | --- |
| `public static final java.lang.String` | `FILE_EXTENSION` | `".xstream"` |

weka.datagenerators.\*

| weka.datagenerators.classifiers.classification.Agrawal | | |
| --- | --- | --- |
| `public static final int` | `FUNCTION_1` | `1` |
| `public static final int` | `FUNCTION_10` | `10` |
| `public static final int` | `FUNCTION_2` | `2` |
| `public static final int` | `FUNCTION_3` | `3` |
| `public static final int` | `FUNCTION_4` | `4` |
| `public static final int` | `FUNCTION_5` | `5` |
| `public static final int` | `FUNCTION_6` | `6` |
| `public static final int` | `FUNCTION_7` | `7` |
| `public static final int` | `FUNCTION_8` | `8` |
| `public static final int` | `FUNCTION_9` | `9` |

| weka.datagenerators.clusterers.BIRCHCluster | | |
| --- | --- | --- |
| `public static final int` | `GRID` | `0` |
| `public static final int` | `ORDERED` | `0` |
| `public static final int` | `RANDOM` | `2` |
| `public static final int` | `RANDOMIZED` | `1` |
| `public static final int` | `SINE` | `1` |

| weka.datagenerators.clusterers.SubspaceCluster | | |
| --- | --- | --- |
| `public static final int` | `CONTINUOUS` | `0` |
| `public static final int` | `GAUSSIAN` | `2` |
| `public static final int` | `INTEGER` | `1` |
| `public static final int` | `TOTAL_UNIFORM` | `1` |
| `public static final int` | `UNIFORM_RANDOM` | `0` |

weka.experiment.\*

| weka.experiment.DatabaseUtils | | |
| --- | --- | --- |
| `public static final int` | `BOOL` | `1` |
| `public static final int` | `BYTE` | `3` |
| `public static final int` | `DATE` | `8` |
| `public static final int` | `DOUBLE` | `2` |
| `public static final java.lang.String` | `EXP_INDEX_TABLE` | `"Experiment_index"` |
| `public static final java.lang.String` | `EXP_RESULT_COL` | `"Result_table"` |
| `public static final java.lang.String` | `EXP_RESULT_PREFIX` | `"Results"` |
| `public static final java.lang.String` | `EXP_SETUP_COL` | `"Experiment_setup"` |
| `public static final java.lang.String` | `EXP_TYPE_COL` | `"Experiment_type"` |
| `public static final int` | `FLOAT` | `7` |
| `public static final int` | `INTEGER` | `5` |
| `public static final int` | `LONG` | `6` |
| `public static final java.lang.String` | `PROPERTY_FILE` | `"weka/experiment/DatabaseUtils.props"` |
| `public static final int` | `SHORT` | `4` |
| `public static final int` | `STRING` | `0` |
| `public static final int` | `TEXT` | `9` |
| `public static final int` | `TIME` | `10` |

| weka.experiment.ResultMatrix | | |
| --- | --- | --- |
| `public static final int` | `SIGNIFICANCE_LOSS` | `2` |
| `public static final int` | `SIGNIFICANCE_TIE` | `0` |
| `public static final int` | `SIGNIFICANCE_WIN` | `1` |

| weka.experiment.TaskStatusInfo | | |
| --- | --- | --- |
| `public static final int` | `FAILED` | `2` |
| `public static final int` | `FINISHED` | `3` |
| `public static final int` | `PROCESSING` | `1` |
| `public static final int` | `TO_BE_RUN` | `0` |

| weka.experiment.xml.XMLExperiment | | |
| --- | --- | --- |
| `public static final java.lang.String` | `NAME_CLASSFIRST` | `"classFirst"` |
| `public static final java.lang.String` | `NAME_PROPERTYNODE_PARENTCLASS` | `"parentClass"` |
| `public static final java.lang.String` | `NAME_PROPERTYNODE_PROPERTY` | `"property"` |
| `public static final java.lang.String` | `NAME_PROPERTYNODE_VALUE` | `"value"` |

weka.filters.\*

| weka.filters.supervised.attribute.ClassOrder | | |
| --- | --- | --- |
| `public static final int` | `FREQ_ASCEND` | `0` |
| `public static final int` | `FREQ_DESCEND` | `1` |
| `public static final int` | `RANDOM` | `2` |

| weka.filters.supervised.attribute.PLSFilter | | |
| --- | --- | --- |
| `public static final int` | `ALGORITHM_PLS1` | `2` |
| `public static final int` | `ALGORITHM_SIMPLS` | `1` |
| `public static final int` | `PREPROCESSING_CENTER` | `1` |
| `public static final int` | `PREPROCESSING_NONE` | `0` |
| `public static final int` | `PREPROCESSING_STANDARDIZE` | `2` |

| weka.filters.unsupervised.attribute.ClassAssigner | | |
| --- | --- | --- |
| `public static final int` | `FIRST` | `0` |
| `public static final int` | `LAST` | `-2` |
| `public static final int` | `UNSET` | `-1` |

| weka.filters.unsupervised.attribute.InterquartileRange | | |
| --- | --- | --- |
| `public static final int` | `NON_NUMERIC` | `-1` |

| weka.filters.unsupervised.attribute.MathExpression | | |
| --- | --- | --- |
| `public static final java.lang.String` | `m_defaultExpression` | `"(A-MIN)/(MAX-MIN)"` |

| weka.filters.unsupervised.attribute.MultiInstanceToPropositional | | |
| --- | --- | --- |
| `public static final int` | `WEIGHTMETHOD_1` | `1` |
| `public static final int` | `WEIGHTMETHOD_INVERSE1` | `2` |
| `public static final int` | `WEIGHTMETHOD_INVERSE2` | `3` |
| `public static final int` | `WEIGHTMETHOD_ORIGINAL` | `0` |

| weka.filters.unsupervised.attribute.RandomProjection | | |
| --- | --- | --- |
| `public static final int` | `GAUSSIAN` | `3` |
| `public static final int` | `SPARSE1` | `1` |
| `public static final int` | `SPARSE2` | `2` |

| weka.filters.unsupervised.attribute.StringToWordVector | | |
| --- | --- | --- |
| `public static final int` | `FILTER_NONE` | `0` |
| `public static final int` | `FILTER_NORMALIZE_ALL` | `1` |
| `public static final int` | `FILTER_NORMALIZE_TEST_ONLY` | `2` |

| weka.filters.unsupervised.attribute.Wavelet | | |
| --- | --- | --- |
| `public static final int` | `ALGORITHM_HAAR` | `0` |
| `public static final int` | `PADDING_ZERO` | `0` |

| weka.filters.unsupervised.instance.subsetbyexpression.Scanner | | |
| --- | --- | --- |
| `public static final int` | `STRING` | `2` |
| `public static final int` | `YYEOF` | `-1` |
| `public static final int` | `YYINITIAL` | `0` |

| weka.filters.unsupervised.instance.subsetbyexpression.sym | | |
| --- | --- | --- |
| `public static final int` | `ABS` | `10` |
| `public static final int` | `AND` | `29` |
| `public static final int` | `ATTRIBUTE` | `34` |
| `public static final int` | `BOOLEAN` | `33` |
| `public static final int` | `CEIL` | `20` |
| `public static final int` | `COMMA` | `2` |
| `public static final int` | `COS` | `15` |
| `public static final int` | `DIVISION` | `9` |
| `public static final int` | `EOF` | `0` |
| `public static final int` | `EQ` | `27` |
| `public static final int` | `error` | `1` |
| `public static final int` | `EXP` | `13` |
| `public static final int` | `FALSE` | `22` |
| `public static final int` | `FLOOR` | `18` |
| `public static final int` | `GE` | `26` |
| `public static final int` | `GT` | `25` |
| `public static final int` | `IS` | `31` |
| `public static final int` | `ISMISSING` | `5` |
| `public static final int` | `LE` | `24` |
| `public static final int` | `LOG` | `12` |
| `public static final int` | `LPAREN` | `3` |
| `public static final int` | `LT` | `23` |
| `public static final int` | `MINUS` | `6` |
| `public static final int` | `NOT` | `28` |
| `public static final int` | `NUMBER` | `32` |
| `public static final int` | `OR` | `30` |
| `public static final int` | `PLUS` | `7` |
| `public static final int` | `POW` | `19` |
| `public static final int` | `RINT` | `17` |
| `public static final int` | `RPAREN` | `4` |
| `public static final int` | `SIN` | `14` |
| `public static final int` | `SQRT` | `11` |
| `public static final int` | `STRING` | `35` |
| `public static final int` | `TAN` | `16` |
| `public static final int` | `TIMES` | `8` |
| `public static final int` | `TRUE` | `21` |

weka.gui.\*

| weka.gui.ConverterFileChooser | | |
| --- | --- | --- |
| `public static final int` | `LOADER_DIALOG` | `1` |
| `public static final int` | `SAVER_DIALOG` | `2` |
| `public static final int` | `UNHANDLED_DIALOG` | `0` |

| weka.gui.GenericObjectEditor | | |
| --- | --- | --- |
| `public static final java.lang.String` | `GUIEDITORS_PROPERTY_FILE` | `"weka/gui/GUIEditors.props"` |

| weka.gui.GenericObjectEditor.GOETreeNode | | |
| --- | --- | --- |
| `public static final java.lang.String` | `MAYBE_SUPPORT` | `"blue"` |
| `public static final java.lang.String` | `NO_SUPPORT` | `"silver"` |

| weka.gui.GenericPropertiesCreator | | |
| --- | --- | --- |
| `public static final java.lang.String` | `USE_DYNAMIC` | `"UseDynamic"` |
| `public static final boolean` | `VERBOSE` | `false` |

| weka.gui.JListHelper | | |
| --- | --- | --- |
| `public static final int` | `MOVE_DOWN` | `1` |
| `public static final int` | `MOVE_UP` | `0` |

| weka.gui.ListSelectorDialog | | |
| --- | --- | --- |
| `public static final int` | `APPROVE_OPTION` | `0` |
| `public static final int` | `CANCEL_OPTION` | `1` |

| weka.gui.LogWindow | | |
| --- | --- | --- |
| `public static final boolean` | `DEBUG` | `false` |
| `public static final java.lang.String` | `STYLE_STDERR` | `"stderr"` |
| `public static final java.lang.String` | `STYLE_STDOUT` | `"stdout"` |

| weka.gui.Main | | |
| --- | --- | --- |
| `public static final int` | `GUI_MDI` | `0` |
| `public static final int` | `GUI_SDI` | `1` |

| weka.gui.PropertySelectorDialog | | |
| --- | --- | --- |
| `public static final int` | `APPROVE_OPTION` | `0` |
| `public static final int` | `CANCEL_OPTION` | `1` |

| weka.gui.SimpleDateFormatEditor | | |
| --- | --- | --- |
| `public static final java.lang.String` | `DEFAULT_FORMAT` | `"yyyy-MM-dd\'T\'HH:mm:ss"` |

| weka.gui.ViewerDialog | | |
| --- | --- | --- |
| `public static final int` | `APPROVE_OPTION` | `0` |
| `public static final int` | `CANCEL_OPTION` | `1` |

| weka.gui.arffviewer.ArffViewerMainPanel | | |
| --- | --- | --- |
| `public static final int` | `DEFAULT_HEIGHT` | `-1` |
| `public static final int` | `DEFAULT_LEFT` | `-1` |
| `public static final int` | `DEFAULT_TOP` | `-1` |
| `public static final int` | `DEFAULT_WIDTH` | `-1` |
| `public static final int` | `HEIGHT` | `600` |
| `public static final int` | `WIDTH` | `800` |

| weka.gui.beans.BeanInstance | | |
| --- | --- | --- |
| `public static final int` | `BEAN_EXECUTING` | `1` |
| `public static final int` | `IDLE` | `0` |

| weka.gui.beans.BeanVisual | | |
| --- | --- | --- |
| `public static final int` | `EAST_CONNECTOR` | `2` |
| `public static final java.lang.String` | `ICON_PATH` | `"weka/gui/beans/icons/"` |
| `public static final int` | `NORTH_CONNECTOR` | `0` |
| `public static final int` | `SOUTH_CONNECTOR` | `1` |
| `public static final int` | `WEST_CONNECTOR` | `3` |

| weka.gui.beans.Classifier | | |
| --- | --- | --- |
| `public static final java.lang.String` | `FILE_EXTENSION` | `"model"` |

| weka.gui.beans.IncrementalClassifierEvent | | |
| --- | --- | --- |
| `public static final int` | `BATCH_FINISHED` | `2` |
| `public static final int` | `NEW_BATCH` | `0` |
| `public static final int` | `WITHIN_BATCH` | `1` |

| weka.gui.beans.InstanceEvent | | |
| --- | --- | --- |
| `public static final int` | `BATCH_FINISHED` | `2` |
| `public static final int` | `FORMAT_AVAILABLE` | `0` |
| `public static final int` | `INSTANCE_AVAILABLE` | `1` |

| weka.gui.beans.KnowledgeFlowApp | | |
| --- | --- | --- |
| `public static final java.lang.String` | `FILE_EXTENSION` | `".kf"` |
| `public static final java.lang.String` | `FILE_EXTENSION_XML` | `".kfml"` |

| weka.gui.beans.SerializedModelSaver | | |
| --- | --- | --- |
| `public static final int` | `BINARY` | `0` |
| `public static final java.lang.String` | `FILE_EXTENSION` | `"model"` |
| `public static final int` | `KOMLV` | `1` |
| `public static final int` | `XSTREAM` | `2` |

| weka.gui.beans.xml.XMLBeans | | |
| --- | --- | --- |
| `public static final int` | `DATATYPE_LAYOUT` | `0` |
| `public static final int` | `DATATYPE_USERCOMPONENTS` | `1` |
| `public static final int` | `INDEX_BEANCONNECTIONS` | `1` |
| `public static final int` | `INDEX_BEANINSTANCES` | `0` |
| `public static final java.lang.String` | `VAL_ANIMATEDICONPATH` | `"animatedIconPath"` |
| `public static final java.lang.String` | `VAL_ASSOCIATEDCONNECTIONS` | `"associatedConnections"` |
| `public static final java.lang.String` | `VAL_BEAN` | `"bean"` |
| `public static final java.lang.String` | `VAL_BEANCONTEXT` | `"beanContext"` |
| `public static final java.lang.String` | `VAL_BLUE` | `"blue"` |
| `public static final java.lang.String` | `VAL_COLOR` | `"color"` |
| `public static final java.lang.String` | `VAL_CUSTOM_NAME` | `"custom_name"` |
| `public static final java.lang.String` | `VAL_DIR` | `"dir"` |
| `public static final java.lang.String` | `VAL_EVENTNAME` | `"eventname"` |
| `public static final java.lang.String` | `VAL_FILE` | `"file"` |
| `public static final java.lang.String` | `VAL_FONT` | `"font"` |
| `public static final java.lang.String` | `VAL_GREEN` | `"green"` |
| `public static final java.lang.String` | `VAL_HEIGHT` | `"height"` |
| `public static final java.lang.String` | `VAL_HIDDEN` | `"hidden"` |
| `public static final java.lang.String` | `VAL_ICONPATH` | `"iconPath"` |
| `public static final java.lang.String` | `VAL_ID` | `"id"` |
| `public static final java.lang.String` | `VAL_INPUTS` | `"inputs"` |
| `public static final java.lang.String` | `VAL_INPUTSID` | `"inputs_id"` |
| `public static final java.lang.String` | `VAL_LOADER` | `"wrappedAlgorithm"` |
| `public static final java.lang.String` | `VAL_LOCATION` | `"location"` |
| `public static final java.lang.String` | `VAL_NAME` | `"name"` |
| `public static final java.lang.String` | `VAL_OPTIONS` | `"options"` |
| `public static final java.lang.String` | `VAL_ORIGINALCOORDS` | `"originalCoords"` |
| `public static final java.lang.String` | `VAL_OUTPUTS` | `"outputs"` |
| `public static final java.lang.String` | `VAL_OUTPUTSID` | `"outputs_id"` |
| `public static final java.lang.String` | `VAL_PREFIX` | `"prefix"` |
| `public static final java.lang.String` | `VAL_RED` | `"red"` |
| `public static final java.lang.String` | `VAL_RELATIONNAMEFORFILENAME` | `"relationNameForFilename"` |
| `public static final java.lang.String` | `VAL_RELATIVE_PATH` | `"useRelativePath"` |
| `public static final java.lang.String` | `VAL_SAVER` | `"wrappedAlgorithm"` |
| `public static final java.lang.String` | `VAL_SIZE` | `"size"` |
| `public static final java.lang.String` | `VAL_SOURCEID` | `"source_id"` |
| `public static final java.lang.String` | `VAL_STYLE` | `"style"` |
| `public static final java.lang.String` | `VAL_SUBFLOW` | `"subFlow"` |
| `public static final java.lang.String` | `VAL_TARGETID` | `"target_id"` |
| `public static final java.lang.String` | `VAL_TEXT` | `"text"` |
| `public static final java.lang.String` | `VAL_WIDTH` | `"width"` |
| `public static final java.lang.String` | `VAL_X` | `"x"` |
| `public static final java.lang.String` | `VAL_Y` | `"y"` |

| weka.gui.boundaryvisualizer.BoundaryPanel | | |
| --- | --- | --- |
| `public static final double` | `REMOVE_POINT_RADIUS` | `7.0` |

| weka.gui.experiment.ExperimenterDefaults | | |
| --- | --- | --- |
| `public static final java.lang.String` | `PROPERTY_FILE` | `"weka/gui/experiment/Experimenter.props"` |

| weka.gui.experiment.OutputFormatDialog | | |
| --- | --- | --- |
| `public static final int` | `APPROVE_OPTION` | `0` |
| `public static final int` | `CANCEL_OPTION` | `1` |

| weka.gui.explorer.ExplorerDefaults | | |
| --- | --- | --- |
| `public static final java.lang.String` | `PROPERTY_FILE` | `"weka/gui/explorer/Explorer.props"` |

| weka.gui.graphvisualizer.GraphConstants | | |
| --- | --- | --- |
| `public static final int` | `DIRECTED` | `1` |
| `public static final int` | `DOUBLE` | `3` |
| `public static final int` | `NORMAL` | `3` |
| `public static final int` | `PLURAL_DUMMY` | `2` |
| `public static final int` | `REVERSED` | `2` |
| `public static final int` | `SINGULAR_DUMMY` | `1` |

| weka.gui.sql.ConnectionPanel | | |
| --- | --- | --- |
| `public static final java.lang.String` | `HISTORY_NAME` | `"connection"` |

| weka.gui.sql.QueryPanel | | |
| --- | --- | --- |
| `public static final java.lang.String` | `HISTORY_NAME` | `"query"` |
| `public static final java.lang.String` | `MAX_ROWS` | `"max_rows"` |

| weka.gui.sql.SqlViewer | | |
| --- | --- | --- |
| `public static final java.lang.String` | `HEIGHT` | `"height"` |
| `public static final java.lang.String` | `WIDTH` | `"width"` |

| weka.gui.sql.event.ConnectionEvent | | |
| --- | --- | --- |
| `public static final int` | `CONNECT` | `0` |
| `public static final int` | `DISCONNECT` | `1` |

| weka.gui.streams.InstanceEvent | | |
| --- | --- | --- |
| `public static final int` | `BATCH_FINISHED` | `3` |
| `public static final int` | `FORMAT_AVAILABLE` | `1` |
| `public static final int` | `INSTANCE_AVAILABLE` | `2` |

| weka.gui.treevisualizer.TreeDisplayEvent | | |
| --- | --- | --- |
| `public static final int` | `ACCEPT` | `3` |
| `public static final int` | `ADD_CHILDREN` | `1` |
| `public static final int` | `CLASSIFY_CHILD` | `4` |
| `public static final int` | `NO_COMMAND` | `0` |
| `public static final int` | `REMOVE_CHILDREN` | `2` |
| `public static final int` | `SEND_INSTANCES` | `5` |

| weka.gui.treevisualizer.TreeVisualizer | | |
| --- | --- | --- |
| `public static final java.lang.String` | `PROPERTIES_FILE` | `"weka/gui/treevisualizer/TreeVisualizer.props"` |

| weka.gui.visualize.Plot2D | | |
| --- | --- | --- |
| `public static final int` | `CONST_AUTOMATIC_SHAPE` | `-1` |
| `public static final int` | `DEFAULT_SHAPE_SIZE` | `2` |
| `public static final int` | `DIAMOND_SHAPE` | `2` |
| `public static final int` | `ERROR_SHAPE` | `1000` |
| `public static final int` | `MAX_SHAPES` | `5` |
| `public static final int` | `MISSING_SHAPE` | `2000` |
| `public static final int` | `PLUS_SHAPE` | `1` |
| `public static final int` | `TRIANGLEDOWN_SHAPE` | `4` |
| `public static final int` | `TRIANGLEUP_SHAPE` | `3` |
| `public static final int` | `X_SHAPE` | `0` |

---


|  |  |  |
| --- | --- | --- |
| **Overview** \| Package \| Class \| **Tree** \| **Deprecated** \| **Index** \| **Help** \| **Weka's home** | |  |
| PREV   NEXT | **FRAMES**    **NO FRAMES**     **All Classes** |  |


---
